# Spinal excitability following sensory electrical stimulation of the upper limb

**DOI:** 10.14814/phy2.70520

**Published:** 2025-09-19

**Authors:** Devin Box, Joshua W. Cohen, Tanya D. Ivanova, Mary Jenkins, Anita Christie, S. Jayne Garland

**Affiliations:** ^1^ School of Kinesiology Western University London Ontario Canada; ^2^ Faculty of Health Sciences Western University London Ontario Canada; ^3^ Department of Clinical Neurological Sciences, Schulich School of Medicine and Dentistry Western University London Ontario Canada; ^4^ School of Physical Therapy Western University London Ontario Canada; ^5^ Department of Physiology and Pharmacology Western University London Ontario Canada

**Keywords:** reciprocal inhibition, spinal excitability, upper limb

## Abstract

The purpose of the study was to determine if sensory electrical stimulation (SES), below motor threshold, would reduce spinal excitability via reciprocal inhibition (RI) and determine if any changes were sex‐related. Eighteen healthy participants (11 males and 7 females) participated in a pre‐post comparison study. The Hoffmann (H‐) reflex was elicited to assess the spinal excitability of Flexor Carpi Radialis (FCR) and the influence of RI from Extensor Carpi Radialis Longus (ECRL) on FCR using a paired conditioning pulse paradigm. A 15‐min bout of SES (4 pulse bursts at 100 Hz) was applied to ECRL, and the H‐reflexes were measured at 0‐ and 20‐min post SES. A linear mixed model regression analysis was performed to evaluate the effects of stimulus order, conditioning, sex, and time on the FCR H‐reflex. All participants experienced RI from the conditioning pulses, with females having significantly greater suppression than males (mean difference; MD = 0.026). For males, SES produced a depression in FCR excitability (MD = 0.023 at time 0; MD = 0.015 at 20 min post‐SES) with no changes in RI. SES had no effect on FCR excitability or RI in females. The potential for SES to produce changes in antagonist excitability was sex‐related, which may have important rehabilitation considerations.

## 1 INTRODUCTION

The excitability of spinal motor neurons is influenced by numerous inputs from cortical or peripheral sources (Lam & Pearson, 2002; Pearson, 2000). Of particular interest is the disynaptic Ia reciprocal inhibition (RI) pathway involving inhibitory interneurons modulated by the antagonistic muscle group. Reciprocal inhibition describes the ability of antagonistic muscle pairs to actively suppress one another (Day et al., 1984), and is integral to the coordinated contraction and relaxation of antagonistic muscle pairs (Crone et al., 1987). Assessment of spinal excitability and the influence of RI can be performed on antagonistic muscle pairs of the upper limb. Such assessment involves evoking a Hoffmann (H‐) reflex in conjunction with an appropriately timed conditioning stimulus applied to the antagonist nerve. The resulting suppression in the H‐reflex amplitude represents the influence of RI through the inhibitory disynaptic Ia pathway (Day et al., 1984).

The role of Ia afferents in the production of use‐dependent spinal plasticity has been proposed as a means of facilitating motor skill learning in a variety of rehabilitation applications (Wolpaw & Tennissen, 2001). Use‐dependent spinal plasticity has been demonstrated in both animal and human studies and suggests that peripheral input could mediate adaptive changes (Heo et al., 2015; McCrea, 2001; Pearson, 2000). Sensory electrical stimulation (SES) applied peripherally has the potential to influence the amount of RI (Milosevic et al., 2019; Obata et al., 2018; Perez et al., 2003). Interestingly, the efficacy of SES‐induced inhibition has been shown to depend on the type of stimulation pattern used (Pascual‐Valdunciel et al., 2023; Perez et al., 2003), stimulation parameters (Bergquist et al., 2011; Dosen et al., 2015), the anatomy of the overlying soft tissue (Kenney et al., 2015), and sex‐related differences (Dornowski et al., 2017).

Among the literature, the lower limbs have been the greater focus of SES research due to more repeatable testing protocols based on favorable anatomy for both stimulating and recording (Mrachacz‐Kersting et al., 2017; Pierrot‐Deseilligny & Mazevet, 2000). Despite this, SES has been used over the past decade as a means to reduce tremor in the upper limb in people with Parkinson's Disease (Heo et al., 2015; Popovic Maneski et al., 2011). Perez et al. (2003) demonstrated that “patterned” SES (delivered as 10 pulses at 100 Hz every 1.5 s) over the common peroneal nerve could increase the reciprocal inhibition effect on the soleus muscle, which lasted after the removal of stimulation. Due to a paucity of research, it is currently unclear whether the underlying spinal circuitry in the upper limb can respond to SES in the same way, and an understanding of this response is crucial to potential upper limb rehabilitation applications like Parkinsonian tremor. In addition, there are important physiological differences between sexes that can influence excitability measurement and delivery of SES (Ansdell et al., 2019; Hunter, 2016; Mendonca et al., 2020). For clinical applicability, it is imperative to understand how sex may influence the response to SES and improve the historical exclusion of females from physiology research (Beery & Zucker, 2011).

The purpose of this study was to examine the effects of patterned SES on the excitability of antagonistic muscle groups in the upper limb and its influence on the RI pathway; a secondary purpose was to examine if any effects were sex‐related. It was hypothesized that the application of SES over the Extensor Carpi Radialis Longus (ECRL) muscle would result in lower excitability of the Flexor Carpi Radialis (FCR) muscle and increased reciprocal inhibition of the FCR, as measured with a conditioned H‐reflex, and persist after the removal of SES. Secondarily, it was hypothesized that the changes would be influenced by sex.

## 2 MATERIALS AND METHODS

### 2.1 Participants

Healthy adult volunteers were recruited from the university community. Inclusion criteria included: (1) being over 18 years of age; (2) having the cognitive capacity to provide consent; (3) being proficient in English; (4) having no prior history of neurological disease or damage. Informed written and verbal consent was acquired from each participant. The study conformed to the standards established by the Declaration of Helsinki latest amendment and was approved by the Institutional Ethics Committee (Research Ethics Board Number: 116007).

Twenty‐five healthy adult volunteers were recruited for this study. During the initial H‐M recruitment curve testing, no stable FCR H‐reflex responses were identified in seven participants (five female and two male); they were excluded from further testing. Of those with H‐reflexes, 11 males and seven females between the ages of 19 and 36 years participated in the study.

### 2.2 Study design

**FIGURE 1:**
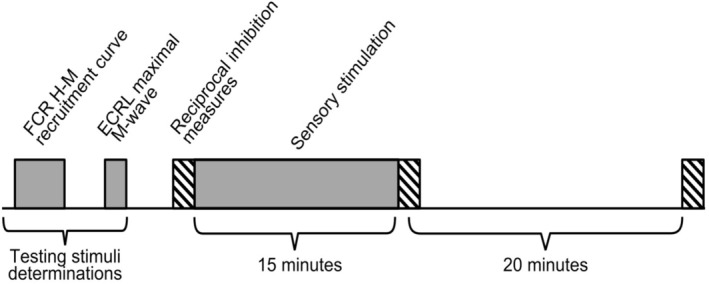
Protocol timeline. Determination of the intensity of test stimuli: H‐M recruitment curve from the flexor carpi radialis (FCR) muscle to find the intensity for the test H‐reflex. Maximal M‐wave from the extensor carpi radialis longus (ECRL) muscle to determine conditioning stimuli intensity. Reciprocal inhibition (RI) measures (striped rectangles) were taken before sensory electrical stimulation (SES) applied for 15 min (Pre), immediately after the SES (Post) and 20 min after SES (Post20).

This study followed a pre/post cross‐sectional comparison design, in which each participant served as their own control. Participants attended a single testing session. Reciprocal inhibition was evaluated prior to (Pre), immediately after (Post), and at 20 min after sensory electrical stimulation (Post20) (Figure 1).

### 2.3 Experimental set up

**FIGURE 2:**
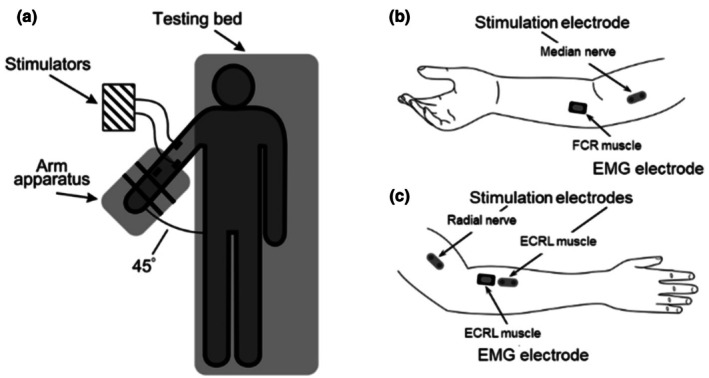
(a) Schematic of the testing setup. Participants laid supine on a testing bed with their right arm abducted at a 45‐degree angle. The right arm was supported by an apparatus on an adjacent testing table. The gap between the two testing tables provided direct access to the stimulating sites on the upper arm. (b and c) Diagrams of recording and stimulating sites on the anterior aspect of the right arm (b), depicting the electrodes used for median nerve stimulation and flexor carpi radialis (FCR) EMG recording, and the posterior aspect of the right arm (c), depicting the electrodes used for radial nerve and extensor carpi radialis longus (ECRL) muscle stimulation, and ECRL EMG recording.

Participants laid supine on a testing bed with their right arm positioned into an apparatus that held the arm at a constant angle of 45 degrees of shoulder abduction and full elbow extension. Participants were instructed to keep their hand completely relaxed during the testing session. A schematic of the setup is shown in Figure 2. Two wireless bipolar surface electromyogram (EMG) electrodes with a 10 mm interelectrode distance were placed on the muscle bellies of the Flexor Carpi Radialis (FCR) and Extensor Carpi Radialis Longus (ECRL) muscles (Trigno Avanti wireless sensors, Delsys Inc., Natick, Massachusetts, USA). The EMG signals (bandwidth: 10‐850 Hz; input range: 22 mV) were recorded using Spike2 v.9.03 software via a Delsys Talker (Cambridge Electronic Design Ltd., Milton, Cambridge, UK) at 2000 Hz.

Electrical stimulation to the median and radial nerves was delivered with a Digitimer DS7A and a Digitimer DS7AH, respectively (Digitimer, Welwyn Garden City, UK) using electrodes that consisted of two stainless steel prongs (~2 mm in diameter) spaced 2.5 cm apart and oriented parallel to the predicted path of the nerve. All stimulating sites are illustrated in Figure 2. The median nerve stimulation electrode was placed along the medial border of the biceps muscle, approximately 1/3 of the length of the humerus proximal to the elbow joint. The radial nerve stimulation site was located on the lateral side of the arm, approximately half of the length of the humerus proximal to the elbow joint between the lateral head of the triceps and the brachialis muscle. The optimal stimulation sites were confirmed by manually triggering the stimulators over the nerve using the same stimulation intensity until the location with highest M‐wave amplitude was found.

**FIGURE 3:**
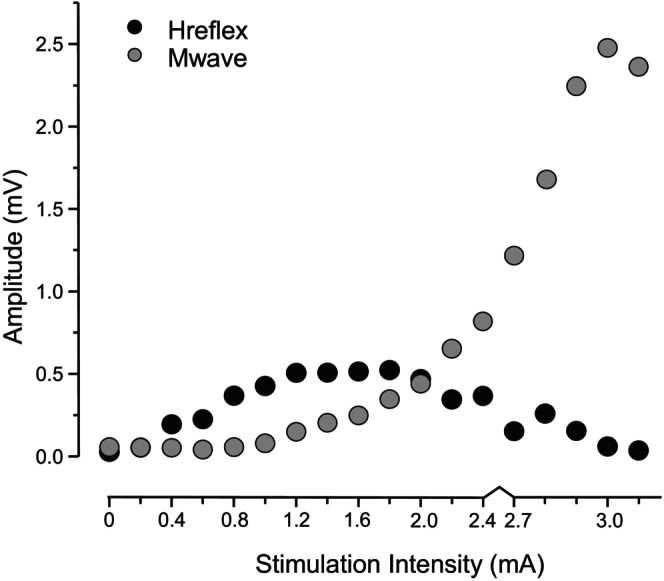
H‐M recruitment curve from a representative participant. Stimulation intensity required for eliciting a maximal H‐reflex (H‐max) and a maximal M‐wave (M‐max) was identified.

At the beginning of the session, a recruitment curve for the H‐reflex and M‐wave (H‐M) from the FCR muscle was obtained (Figure 3). Single‐pulse stimuli of 1 ms duration were delivered to the median nerve every 8 s with increasing intensity from zero until a maximal M‐wave amplitude was achieved. A minimum of 10 stimulations were given over the entire H‐M recruitment range. During the recruitment curve, H‐max and M‐max were identified, and the corresponding stimulation intensity was noted. Following the FCR H‐M recruitment curve, the ECRL M‐max was obtained using 200 μs single‐pulse stimulations. Both M‐max values for FCR and ECRL were confirmed using a supramaximal (120%) stimulation intensity.

### 2.4 Evaluation of reciprocal inhibition

Reciprocal inhibition can be evaluated from the depression of a test H‐reflex combined with an appropriately timed conditioning stimulus applied to the antagonist nerve (Crone et al., 1987). Reciprocal inhibition was assessed by the simultaneous application of FCR test stimulus and ECRL conditioning stimulus (conditioned H‐reflex). Simultaneous application of stimuli was used to maximize the RI and was confirmed with pilot testing. This was alternated with recording the FCR H‐reflex alone to assess unconditioned excitability (unconditioned H‐reflex). Ten pairs of unconditioned/conditioned H‐reflexes were elicited, with at least 8 s between H‐reflexes. Ten pairs were chosen to increase the reliability of the measurement while reducing the total time of testing to ensure that the effect of SES would not wear off in the post SES testing.

Conditioning stimulation intensity of the radial nerve was maintained between 10% and 15% of M‐max for the ECRL muscle (Day et al., 1984). Median nerve testing stimuli were delivered at an intensity used at the ascending part of the H‐reflex recruitment curve, just prior to the H‐max value. Once established, the stimulation intensity was not changed. An FCR M‐max was elicited after each group of 10 pairs of unconditioned/conditioned H‐reflexes to evaluate if any muscle fatigue occurred during the testing session. Stimulation intensities are reported in Table 1.

**TABLE 1:** Stimulus Intensities in mA.

	FCR Mmax	ECRL Mmax	Median testing	Radial testing
Mean ± SD	13.38 ± 10.70	163.06 ± 50.82	5.32 ± 4.34	79.94 ± 26.81
Range	2.43–41.70	85.00–250.00	0.90–15.00	38.50–120.00

### 2.5 Sensory stimulation protocol

A Digitimer DS7A constant current stimulator was used to administer sensory electrical stimulation to the ECRL muscle via a disposable bipolar silver/silver chloride surface electrode with a diameter of 1 cm and an interelectrode distance of 2 cm. This electrode was placed just distal to the ECRL EMG electrode, oriented in parallel to the direction of the muscle fibers.

Stimulation parameters were chosen to maximize Ia afferent recruitment, and similar to those administered in the tremor suppression literature (Bergquist et al., 2011; Dosen et al., 2015). Accordingly, the SES consisted of 4‐pulse bursts (1 ms pulse width at 100 Hz frequency) delivered at 4.6 Hz (i.e., a burst of 31 ms duration was delivered every 217 ms). The duration of the SES session was set at 15 min, delivering a total of 16,560 stimulations. For its applicability in rehabilitation, the pulse amplitude for the SES was set to the same subjective comfort level for all participants. This level was found by achieving a motor response (visually observed muscle twitch), followed by incremental 1 mA decreases until the participant verbally rated the intensity of the sensation during stimulation as a 3 on a 10‐point numerical rating scale (Downie et al., 1978).

### 2.6 Data and statistical analysis

Maximal M‐waves from ECRL and FCR muscles, and H‐reflexes from FCR were measured as peak‐to‐peak amplitude. Maximal M‐waves were analyzed and reported as absolute values (in mV), while the H‐reflexes were normalized to the corresponding maximal M‐wave and are reported as a ratio of the M‐max. RI was evaluated by subtracting the amplitude of the conditioned H‐reflex from that of the unconditioned (test) H‐reflex for each pair of stimuli.

Statistical analyses were conducted using SPSS v.29 (IBM Corp, Armonk, NY) and R (v.5.12.1, R Development Core Team, 2009). To examine the effects of stimulus order (ranging from 1 to 10 unconditioned or conditioned stimulations), conditioning type (unconditioned vs. conditioned), sex (female and male), and time (pre, post, and post20) on both the FCR H‐reflex and M‐max, separate linear mixed‐effects models were constructed utilizing the lme4 package (Ansdell et al., 2019). The linear mixed‐effects modeling approach was chosen to account for variability among participants in the dependent measures. The alpha level for the statistical tests was set to *p* = 0.05.

Model fitting began with a maximal random‐effects structure, incorporating both by‐participant and by‐item random intercepts and slopes. This structure was then iteratively refined to achieve the best‐supported model based on the data, with model comparisons performed using likelihood ratio tests (LRTs). The final model retained by‐participant random intercepts along with random slopes for Stimulus Order and Time, as including random slopes for Conditioning or Sex led to singular fit issues due to collinearity. Model assumptions of normality and homoscedasticity were assessed by inspecting residual diagnostic plots, and all dependent variables were confirmed to meet these assumptions. Two separate linear mixed‐effects models were used to analyze the primary outcomes, M‐max and H‐reflex. In the M‐max model, only Sex and Time were included as independent variables, since Stimulus Order and Conditioning were not relevant for this outcome.

The statistical significance of fixed effects was evaluated using type III Wald *F*‐tests with Kenward‐Roger degrees of freedom, implemented via the ANOVA function in R's car package (v.3.0.12). Post hoc comparisons were performed using Bonferroni‐corrected pairwise contrasts through the estimated‐means approach available in the emmeans package (v.1.7.2) between the levels of conditioning type (unconditioned and conditioned), sex (female and male), and time (Pre, Post, and Post20) averaged over the levels of: Stimulus Order. Confidence intervals for parameter estimates were derived using parametric bootstrapping with 5000 iterations. Post hoc findings are presented with the mean estimate difference (MD), 95% confidence intervals (CIs), and adjusted *p* values.

Finally, a correlation analysis was performed to determine if the change in RI was related to the initial spinal excitability. As the distributions of RI and the change in RI values were not normal, a Spearman rank correlational analysis was performed to assess the relationship between baseline RI and changes in RI post SES.

## 3 RESULTS

**FIGURE 4:**
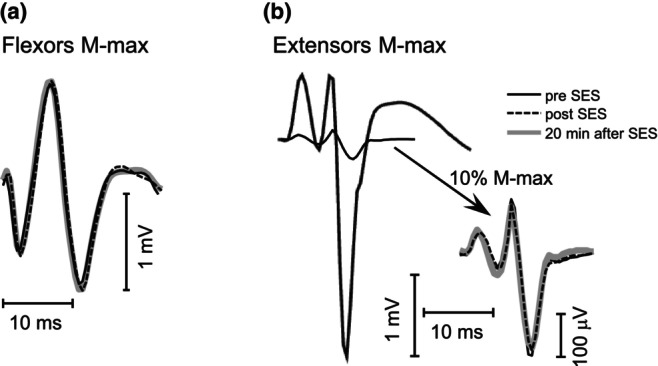
Flexor carpi radialis (FCR) and extensor carpi radialis longus (ECRL) maximal M‐waves from a single participant. (a) FCR M‐max before sensory electrical stimulation (SES) (Pre; solid black line), after SES (Post; dotted black line) and 20 min after SES (Post20; gray line). (b) Maximal ECRL M‐wave, obtained at the beginning of the experiment (thicker black line), used to find the stimulation intensity for conditioning stimulus (approximately 10% of M‐max). The antagonist M‐wave (expanded, right) elicited by the conditioning stimulus did not change in amplitude during Pre (solid black line), Post (dotted black line) or Post20 (gray line).

The analysis of the FCR M‐max showed no difference before and after SES (Figure 4). The estimated means of the FCR M‐max value can be found in Table 2. There were no statistically significant effects of Sex (*F* = 1.87, *p* = 0.08, df = 16) or Time (*F* = 0.9, *p* = 0.64, df = 1035) on the FCR M‐max value, suggesting that the protocol was not fatiguing. There was no effect of stimulus order on the H‐reflex, showing that the testing stimuli themselves did not influence excitability within the Pre, Post, and Post20 testing sessions.

**TABLE 2:** Flexor Carpi Radialis M‐max during testing periods.

Sex	Testing period	M‐max (mV)		
		Mean	CI lower bound	CI upper bound
Female	Pre	1.61	0.58	2.63
	Post	1.70	0.66	2.75
	Post20	1.63	0.62	2.65
Male	Pre	2.97	2.05	3.69
	Post	2.76	1.93	3.59
	Post20	2.76	1.95	3.57

*Note*: M‐max, maximal M‐wave; Pre, before sensory electrical stimulation (SES); Post, immediately after the SES; Post20, 20 min after SES. Data are estimated marginal means ± standard error (SE) and 95% confidence intervals (CI) of the mean.

**FIGURE 5:**
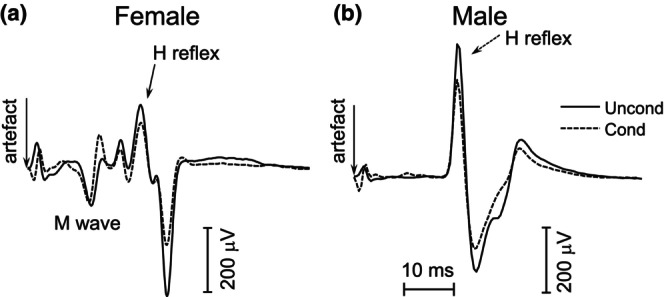
Unconditioned and conditioned H‐reflexes from a female (a) and a male (b) participant. Note the larger change in peak‐to‐peak amplitude in the conditioned H‐reflex in the female participant.

**FIGURE 6:**
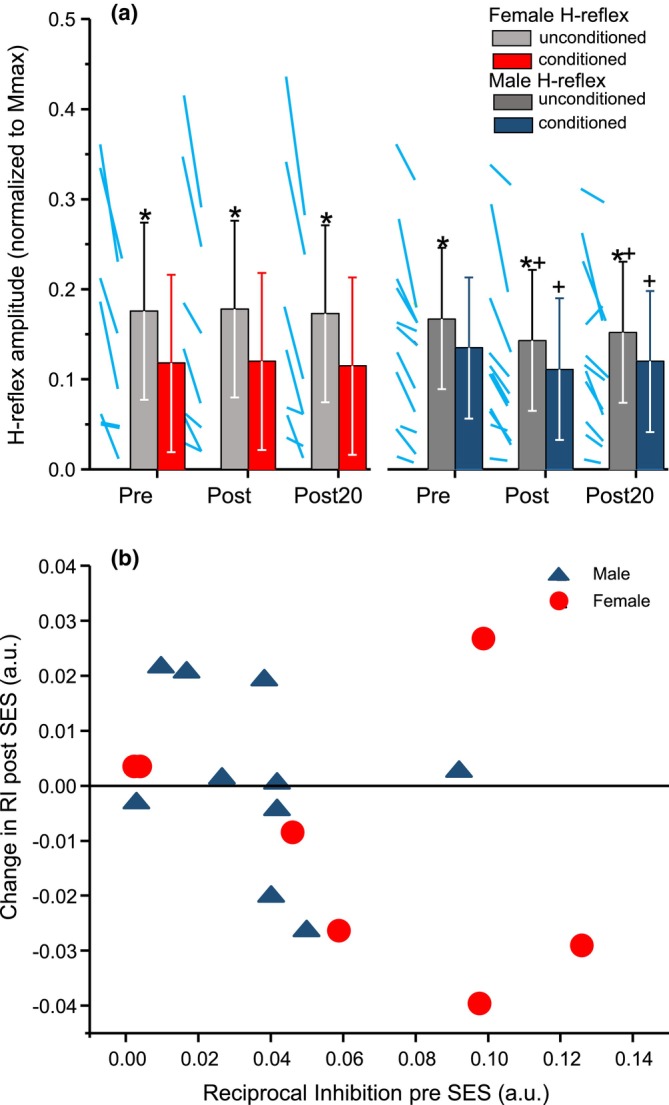
(a) Unconditioned and conditioned H‐reflexes at Pre, Post, Post20 for female (left) and male (right) participants. In both panels the average for each participant (Unconditioned to Conditioned) at each time point is shown with blue lines. The bars represent the mean with 95% confidence intervals for unconditioned (light gray—females; dark gray—males) and conditioned (red—females; dark blue—males) H‐ reflexes. * Denotes statistically significant difference from conditioned H‐reflex; + denotes statistically significant difference from Pre testing. (b) The change in reciprocal inhibition (RI) plotted against the Pre RI (before sensory electrical stimulation (SES)) for female (red circles) and male (dark blue triangles) participants. The data for each participant are averages of RI and the change of RI calculated between the pairs of unconditioned and conditioned H‐reflexes pre and post SES. Values below the horizontal line indicate less RI post SES than Pre.

The conditioning stimulus was effective in evoking reciprocal inhibition. Figure 5 depicts the effects of the conditioning stimulus on the peak‐to‐peak amplitude of the H‐reflex. The estimated means, along with individual data, of the H‐reflex amplitudes can be found in Figure 6a. The amount of reciprocal inhibition at baseline varied across participants (4.2%–75.9% of the unconditioned H‐reflex). Correlational analysis showed a statistically significant moderate negative correlation (*p* = −0.52, *p* < 0.001) between changes in RI and baseline suppression values (Figure 6b). That is, the greater the baseline level of RI, the smaller the change in RI after SES.

The H‐reflex amplitude was dependent on the Conditioning (*F =* 15.97, *p* < 0.0001, df = 1035) and had an interaction effect based on Sex Conditioning × Sex interaction (*F* = 5.64, *p* < 0.0001, df = 1035). The post hoc analysis determined that both groups had significant decreases during the conditioned protocol at all time points (Females: MD = 0.058, *p* < 0.0001, df = 1035, Males: MD = 0.032, *p* < 0.0001, df = 1035). However, females had a larger reduction between the unconditioned and conditioned protocol (Figure 5), compared to males (MD = 0.026, *p* = 0.048, df = 16), signifying greater reciprocal inhibition at all time points.

There was no effect of SES on the amount of reciprocal inhibition. The H‐reflex amplitude was dependent on the interaction between Sex and Time (*F* = 4.536, *p* < 0.001, df *= 1035*). Post hoc analysis determined that both the unconditioned and conditioned H‐reflexes were significantly depressed in males immediately following SES (Post: MD = 0.023, *p* < 0.0001, df *= 1035*) and remained significantly lower than the Pre‐testing value 20 min after SES (Post 20: MD = 0.015, *p* < 0.0026, df *= 1035*), signifying a reduction in spinal inhibition without a change in RI. In the females, the conditioned H‐reflex depression was relatively constant over time and did not reach a statistical significance (*p* = 0.81, df *= 1035*) signifying no change in either spinal excitability or RI after SES.

## 4 DISCUSSION

Spinal excitability of the FCR muscle group and the amount of RI created with a conditioning stimulus were assessed before and after a bout of patterned SES. The main findings of the study were that the effect of SES on spinal excitability, as determined by the unconditioned H‐reflex amplitude, was greater in males than in females; whereas, the effect of SES on the amount of reciprocal inhibition, as determined by the conditioned H‐reflex, did not change after SES in either sex. Females demonstrated more reciprocal inhibition at all time points than males.

### 4.1 SES decreases spinal excitability in males

Males experienced a proportional decrease in both conditioned and unconditioned H‐reflex responses post SES, while females did not. Interestingly, in males, the unconditioned H‐reflex showed a significant decrease after SES, suggesting that SES applied to the antagonistic muscle group (ECRL) influences the excitability of the motoneuron pool of the agonist group (FCR) distinct from reciprocal inhibition. Activation of cutaneous mechanoreceptors (or their afferents) has been shown to produce transient inhibition, via segmental or propriospinal pathways (Lourenço et al., 2007). Our findings differ from Milosevic et al. (2019) who found no change in spinal reflex excitability with SES. However, the authors speculated that the stimulation intensities/durations used in their study may not have been strong enough to induce plasticity. Although our study selectively investigated sensory level electrical stimulation (i.e., below the threshold to induce motor response), the duration of our SES was far greater (15 min vs. 1 min) than the comparable literature (Milosevic et al., 2019). We will explore the effect of stimulation intensity when we discuss the lack of effect in females.

### 4.2 Reciprocal inhibition did not change after SES

Contrary to our hypothesis, reciprocal inhibition did not change following SES. Pascual‐Valdunciel et al. (2023) recently demonstrated the ability to produce either an increase or decrease in RI levels in the upper limb when utilizing SES in‐phase or out‐of‐phase to voluntary arm movements, respectively. In their study, priming stimuli were given in phase to voluntary movements that would act synergistically with movement direction, and consequently, would be received by the Ia afferents during their silent period. The lack of voluntary movement incorporated into our study could explain why changes in RI were not found. It is possible that sensitization of the group Ia fibers in the ECRL might only be achievable while voluntary contraction silences physiologic nerve signaling and minimizes antidromic collisions. This is supported by the fact that out‐of‐phase stimulation is thought to produce decreases in RI due to antidromic collisions from simultaneous stimulation and physiologic Ia activity (Formento et al., 2018). The robustness of this effect is better demonstrated in the lower limb. Perez et al. (2003) showed that SES can induce greater RI in the lower limb but only when applied in a pattern mimicking the gait cycle. Patterned SES alone appears to have the potential to induce robust plasticity in the inhibitory interneuron pathway of the lower limb lasting after the application of stimulation is removed. Taken together, both studies highlight that SES may be very specific to movement patterns or voluntary coactivation.

### 4.3 Sex‐related differences

Our results show a significant interaction between sex and time, revealing that the reduction in H‐reflex excitability was only seen in males and not females. Known differences exist between the sexes in muscle size and fiber type composition (Hunter, 2016), endurance and oxidative capacity (Ansdell et al., 2019), and differences in the Hmax:Mmax ratio (Mendonca et al., 2020).

Given that our study set the SES intensity at a subjective pain level, it is entirely possible that females did not receive enough total stimulation to induce plasticity. Differences in the tolerability of SES can be influenced by many factors outside of those collected in the present study, including participant hydration, subcutaneous fat content, and the type of electrodes used (Keller & Kuhn, 2009; Lyons et al., 2004; Vance et al., 2015). The goal of any SES regimen is to selectively activate primary sensory afferents; however, electrical currents will activate pain receptors on the skin surface. With increasing stimulation intensities, activation of pain receptors can produce discomfort, ultimately limiting the effectiveness of SES regimens (Gracanin & Trnkoczy, 1975). Because females typically have greater fat content at the stimulating sites (Olarogba et al., 2024), a greater proportion of the stimulation current could have been picked up by pain receptors, rather than primary sensory afferents; a common effect described as dispersion (Burke et al., 1999). Consequently, our participants' rating of subjective pain during the SES session, which was selected for its applicability to rehabilitation interventions, could be influenced by this dispersion effect. Additionally, our finding of a negative correlation between initial RI and change in RI post SES implies that the amount of initial RI reduces the magnitude of RI change. Given that females had higher baseline RI, this would suggest a smaller overall potential for change on average. These sex‐related differences may be an important consideration when standardizing SES regimens in the upper limb for tremor.

Our finding of greater RI in females appears to stand in contrast with previous literature. In the lower limb, female participants have been shown to produce greater levels of antagonist co‐activation during submaximal and maximal force levels (De Luca & Mambrito, 1987; Nielsen & Kagamihara, 1992). One of the mechanisms underlying this difference is thought to be depressed levels of reciprocal inhibition, consequently allowing greater antagonist force production. Because our study was performed at rest, it is possible that this could explain our findings of greater reciprocal inhibition in females. However, given the scarcity of literature comparing RI between sexes in the upper limb, no strong conclusion can be drawn from our findings alone.

### 4.4 Limitations

Limitations of our study include the fact that this was a study with a relatively small sample size performed on young healthy individuals. Our results show considerable variability in response to SES. This variability may have resulted from the intensity of the stimulation (relative to each participant's comfort level). This intensity level was chosen to approximate how clinicians might titrate the intensity of SES in clinical applications. Additionally, there was a significant decrease in the amplitude of the unconditioned H‐reflex following SES in male participants; therefore, we cannot rule out a reduction in excitability of the H‐reflex pathway in males that may have influenced the calculation of RI. However, we do not view this as a major limitation, as RI is calculated as a relative change in H‐reflex amplitude, which did not change post SES.

## 5 CONCLUSIONS

Our results suggest that SES has the potential to influence the excitability of the spinal motor neurons of the antagonist muscle group in homonymous limbs. In males, plasticity was achieved through a reduction in spinal excitability, rather than reciprocal inhibition. These findings could have implications for further research investigating SES in rehabilitation settings.

## AUTHOR CONTRIBUTIONS

DB, TI, JG, MJ, and AC were involved in the conception and design of the research study. DB, TI, and JG were involved in data collection. TI and JC were involved in data analysis and figure preparation. DB drafted the manuscript. TI, JG, MJ, AC, and JC edited and revised the manuscript for final submission.

## FUNDING INFORMATION

This research was supported by the Natural Sciences and Engineering Research Council of Canada grant (GPIN 105424‐12).

## CONFLICT OF INTEREST STATEMENT

The authors have no real or perceived conflicts of interest to disclose relating to the work presented herein.

## ETHICS STATEMENT

The study conformed to the standards established by the Declaration of Helsinki latest amendment and was approved by the Institutional Ethics Committee (Research Ethics Board Number: 116007).

## Data Availability

Data will be available upon request.
